# Recurrent postoperative pulmonary hemorrhage complicated by asphyxia-induced cardiac arrest successfully managed with extracorporeal membrane oxygenation in an infant: a case report

**DOI:** 10.3389/fped.2026.1777946

**Published:** 2026-03-16

**Authors:** Juho Yoneshige, Shota Sonobe, Ryohei Fukuba, Rei Tonomura, Nobuyuki Tsujii, Takahiro Kajimoto, Junji Egawa, Masahiko Kawaguchi

**Affiliations:** 1 Department of Anesthesiology, Nara Medical University, Kashihara, Japan; 2 Department of Anesthesiology, Nagoya University Hospital, Nagoya, Japan; 3 Department of Thoracic and Cardiovascular Surgery, Nara Medical University, Kashihara, Japan; 4 Department of Pediatrics, Nara Medical University, Kashihara, Japan

**Keywords:** bronchial blocker catheter, congenital heart disease—cardiac, extracorporeal membrane oxygenation (ECMO), infant, major aortopulmonary collateral arteries (MAPCAs), pulmonary hemorrhage

## Abstract

This report describes an infant who experienced two distinct episodes of postoperative pulmonary hemorrhage after a Rastelli-type procedure, each driven by a different mechanism requiring tailored management. The initial right-sided pulmonary hemorrhage was attributed to excessive flow through major aortopulmonary collateral arteries (MAPCAs). Although hemostasis could not be achieved by coil embolization alone, selective temporary occlusion using balloon catheter limited the bleeding to a more segmental distribution. Despite airway obstruction leading to cardiac arrest, bleeding did not worsen under veno-arterial extracorporeal membrane oxygenation (VA-ECMO), which allowed stabilization and ultimately resulted in full recovery. The subsequent left-sided pulmonary hemorrhage was likely caused by elevated left atrial pressure following moderate residual atrioventricular valve regurgitation. Decompression with temporary left atrial venting, followed by atrial septal defect (ASD) creation, resulted in marked improvement. This case illustrates that VA-ECMO can be lifesaving even in pediatric cardiac arrest caused by massive pulmonary hemorrhage when ventilation becomes impossible. It also highlights that left atrial decompression—including ASD creation—can be an effective therapeutic strategy for pulmonary hemorrhage driven by elevated left atrial pressure. Furthermore, it suggests that selective balloon occlusion may help localize and attenuate bleeding in MAPCA-related pulmonary hemorrhage.

## Introduction

Pulmonary hemorrhage after congenital heart surgery is rare but may rapidly cause airway obstruction, severe hypoxemia, and pulmonary hemorrhage–related asphyxia leading to cardiopulmonary collapse (1). Although veno-arterial extracorporeal membrane oxygenation (VA-ECMO) is an established rescue modality for postoperative cardiopulmonary failure, its use in the presence of active pulmonary hemorrhage remains highly controversial because systemic anticoagulation required during ECMO has been consistently associated with increased bleeding risk, including pulmonary hemorrhage (2). As a result, published reports describing ECMO initiation specifically for cardiopulmonary arrest caused by pulmonary hemorrhage–related asphyxia are exceedingly scarce (1, 2). Against this background, we report an infant who experienced two discrete episodes of postoperative pulmonary hemorrhage following a Rastelli-type repair—each driven by a different mechanism. The first episode resulted in pulmonary hemorrhage–related asphyxia and cardiac arrest during active airway bleeding, yet VA-ECMO was safely initiated without worsening hemorrhage. The second episode, caused by elevated left atrial pressure from severe residual left atrioventricular valve regurgitation, was successfully controlled by left atrial decompression through atrial septal defect creation.

This case highlights a rarely documented but clinically important scenario: even in the setting of ongoing pulmonary hemorrhage, ECMO can be lifesaving when cardiopulmonary arrest is primarily attributable to pulmonary hemorrhage–related asphyxia rather than hemorrhagic shock.

## Case presentation

**Figure 1:**
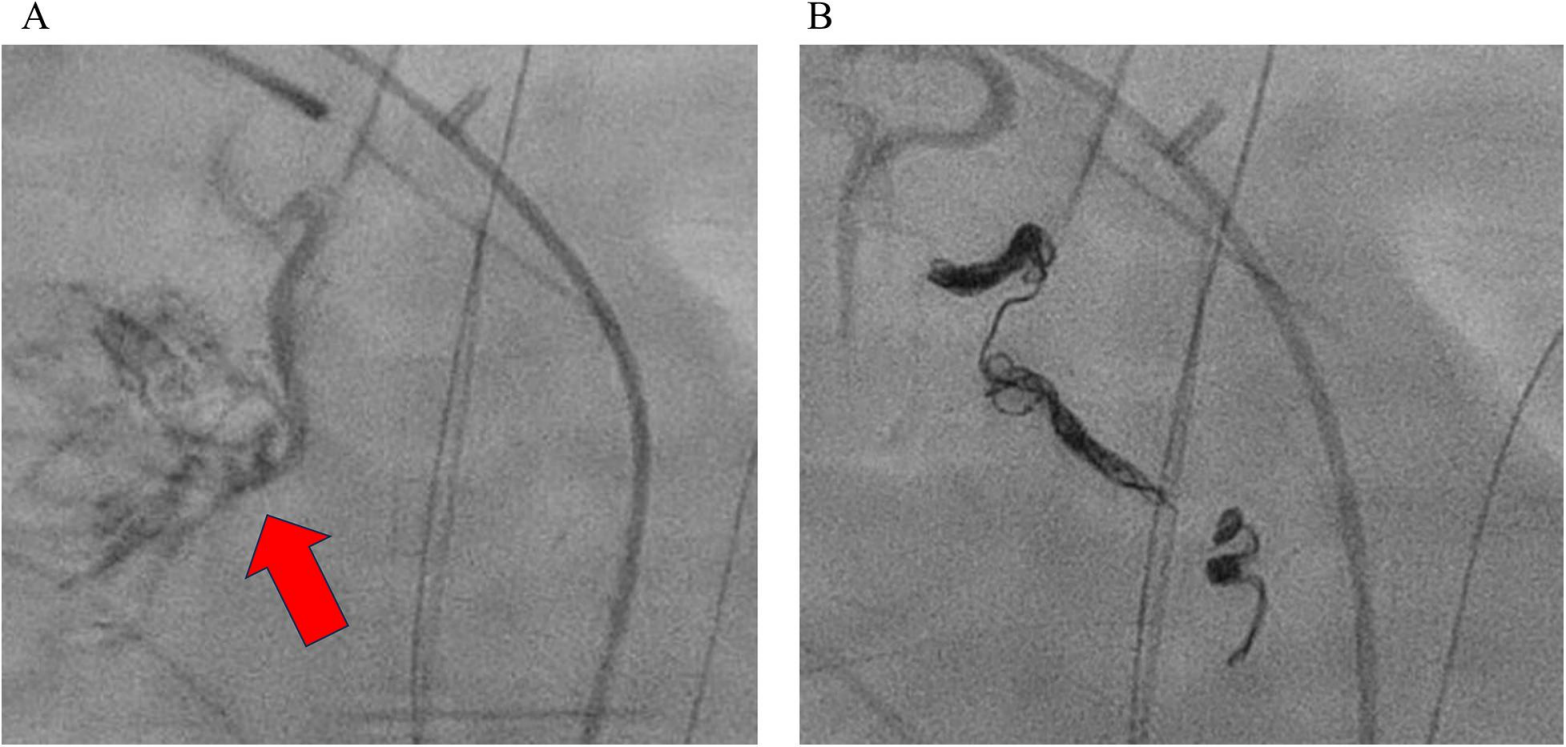
Contrast angiography demonstrating diffuse extravasation from MAPCAs into the lung parenchyma **(A)**. In the interventional radiology suite, simultaneous bronchoscopic compression hemostasis and transcatheter embolization of MAPCAs arising from the right subclavian artery and the descending aorta were performed **(B)**.

**Figure 2:**
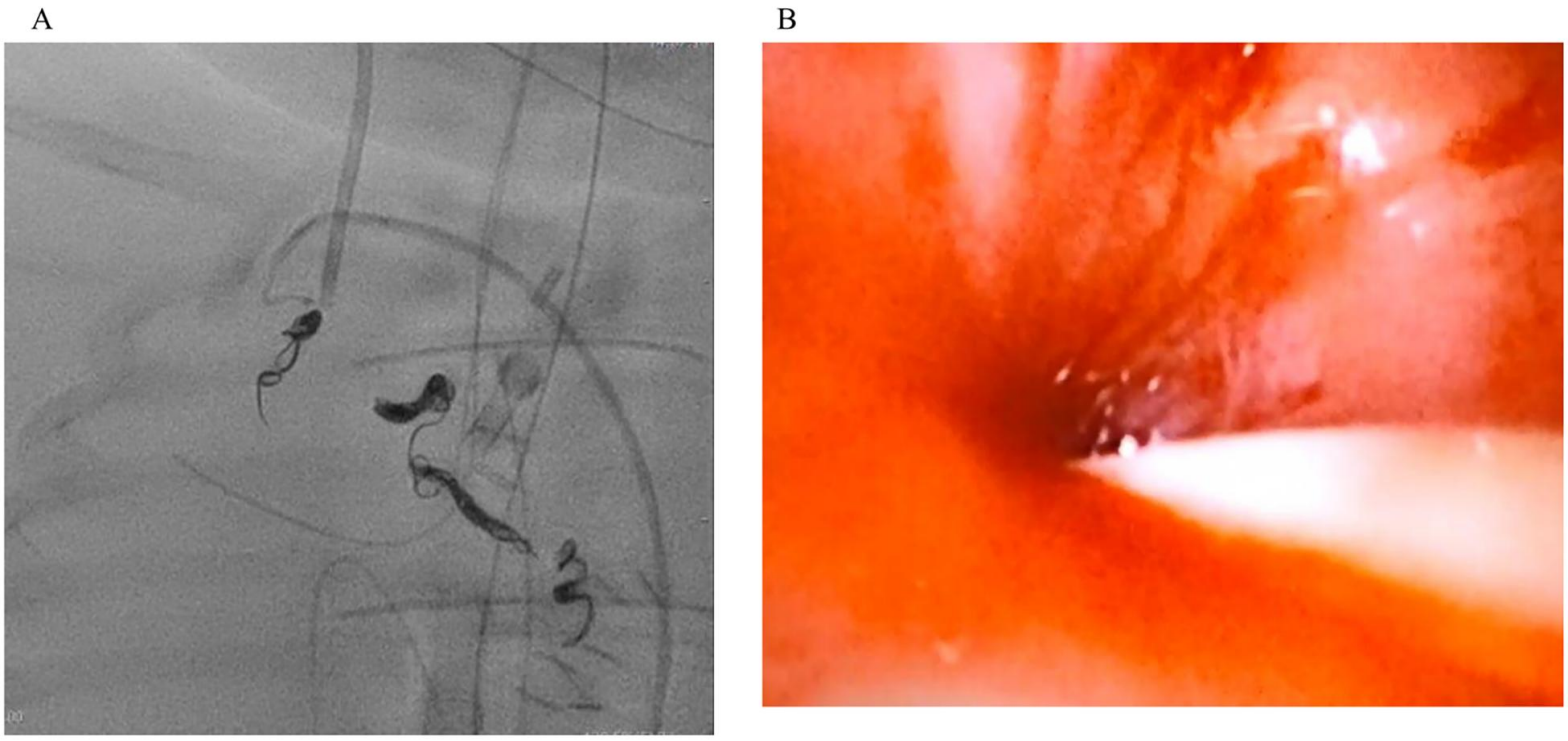
Repeat angiography demonstrating persistent bleeding from residual MAPCAs **(A)**. Under bronchoscopic visualization, transcatheter coil embolization of MAPCAs arising from the right internal thoracic artery and the descending aorta was performed, while a bronchial blocker was used to achieve local airway control. Hemostasis was subsequently confirmed by bronchoscopy **(B)**.

**Figure 3:**
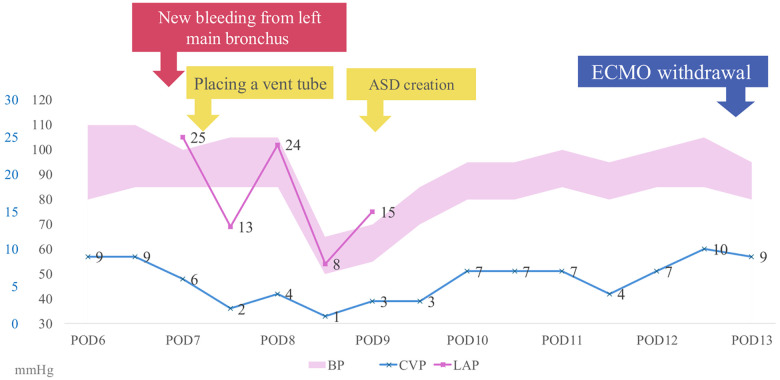
Time course of left atrial pressure (LAP), central venous pressure (CVP), and blood pressure (BP) during the second episode of pulmonary hemorrhage. On postoperative day (POD) 7, new left-sided pulmonary bleeding was observed in association with elevated LAP. Placement of a left atrial (LA) vent on POD 8 reduced LAP from 25 to 13 mmHg, followed by ASD creation and subsequent stabilization, allowing successful withdrawal of extracorporeal membrane oxygenation (ECMO).

A 5-month-old male infant weighing 3.7 kg was admitted for elective definitive repair of congenital heart disease. The patient had been diagnosed with VACTERL association, tetralogy of Fallot, partial atrioventricular septal defect, and major aortopulmonary collateral arteries (MAPCAs). In early infancy, a modified- Blalock-Taussig shunt was performed as an initial palliative procedure. After subsequent evaluation confirming suitability for definitive repair, a Rastelli-type procedure was planned and performed. Intraoperatively, previously unrecognized straddling of the right atrioventricular valve chordae into the left ventricle was identified. Because reconstruction of this abnormal chordal attachment was technically difficult, moderate residual left atrioventricular valve regurgitation (LAVVR). This residual regurgitation was considered to have caused an acute elevation in left atrial pressure (LAP). On postoperative day (POD) 2, the patient developed sudden-onset massive right-sided pulmonary hemorrhage. Bronchoscopy confirmed active bleeding from the right lung. Ventilatory management with continuous muscle relaxation and high levels of PEEP temporarily improved oxygenation; however, bleeding persisted. Given the preoperative presence of MAPCAs, excessive MAPCA flow was suspected as the main etiology. On POD 4, the hemorrhage worsened, and angiography demonstrated significant MAPCA flow originating from the right subclavian artery and descending aorta (Figure 1A). Coil embolization was performed (Figure 1B), and a 2 Fr bronchial blocker was placed to achieve temporary hemostasis. Shortly after returning to the intensive care unit following coil embolization, the patient suddenly developed recurrent massive pulmonary bleeding. The hemorrhage rapidly flooded the central airway, resulting in acute airway obstruction, severe hypoxia, and subsequent cardiac arrest. Given the open-chest postoperative state, central VA-ECMO was promptly established via direct cannulation of the great vessels, leading to successful return of spontaneous circulation. Because systemic anticoagulation required for ECMO carries a risk of exacerbating active bleeding, anticoagulation was managed with extreme caution. An initial intravenous bolus of unfractionated heparin at 135 U/kg (total dose: 500 U) failed to produce a significant prolongation of activated clotting time (ACT); therefore, an additional 200 U was administered. Subsequently, central VA-ECMO was initiated, and anticoagulation was maintained using nafamostat mesylate, targeting an ACT of ≥180 s. On POD 5, angiography revealed residual MAPCA flow, and additional embolization procedures were performed (Figures 2A,B). Hemostasis was confirmed on POD 6, allowing removal of the bronchial blocker. On POD 7, a new episode of left-sided pulmonary hemorrhage occurred. Angiography confirmed minimal left-sided MAPCA flow, and echocardiography suggested that the new hemorrhage might be related to elevated LAP secondary to severe LAVVR. A left atrial vent was placed, reducing LAP from 25 to 13 mmHg (Figure 3), which stabilized the bleeding. On POD 8, hemolysis due to ECMO circuit obstruction occurred. To optimize left atrial decompression, atrial septal defect (ASD) creation was performed, followed by mitral valve repair. Hemodynamics improved gradually, and ECMO was successfully withdrawn on POD 13. Mechanical ventilation was discontinued on POD 31, and the patient was transferred from the intensive care unit to the general ward on POD 36. No apparent neurological abnormalities were observed during the remainder of the hospital course.

## Discussion

Because pulmonary hemorrhage occurred in two distinct episodes, the underlying pathophysiology of each event warrants careful consideration. The first episode was most likely caused by acute pulmonary congestion resulting from a sudden elevation in LAP, which was attributable to a rapid increase in left atrioventricular valve regurgitation combined with excessive pulmonary blood flow from MAPCAs. In contrast, the second episode was interpreted as diffuse pulmonary hemorrhage primarily driven by persistently elevated LAP. Accordingly, surgical interventions aimed at reducing LAP—including insertion of a left atrial vent, creation of an ASD, and re-plasty of the left atrioventricular valve—were performed to address the fundamental pathophysiology. There have been several reports describing the use of ECMO in the setting of pulmonary hemorrhage, particularly when complicated by refractory hypoxemic respiratory failure (1–7). However, most previously reported cases involved underlying respiratory failure, hemodynamic compromise, or other primary diseases. In contrast, our case is unique in that the patient developed pulmonary hemorrhage followed by airway obstruction, asphyxia, and ultimately cardiac arrest—a sequence that has been rarely documented (8). The greatest strength of this case is that ECMO was successfully initiated despite this complex presentation, resulting in complete neurological recovery. In general, clinicians often hesitate to introduce ECMO during active hemorrhage because of concerns that anticoagulation may worsen bleeding (2). We experienced the same hesitation in this case. In pediatric ECMO at our institution, unfractionated heparin is routinely used as standard systemic anticoagulation. However, in the present case, the patient had ongoing and massive pulmonary hemorrhage at the time of ECMO initiation, raising significant concern that conventional heparinization could exacerbate bleeding. For this reason, we selected nafamostat mesylate as an alternative anticoagulant because of its extremely short half-life and reported lower bleeding risk, which allowed more flexible anticoagulation control during active hemorrhage. We also considered initiating ECMO without systemic anticoagulation, an approach that has been described in select cases with severe bleeding. However, given the anticipated duration of ECMO support and the high risk of circuit thrombosis in this infant, we judged that complete avoidance of anticoagulation would be unsafe. Therefore, a low-intensity anticoagulation strategy using nafamostat was adopted as a compromise between bleeding control and circuit patency. This individualized decision-making process underscores the controversial and case-dependent nature of anticoagulation management during ECMO in patients with active pulmonary hemorrhage.

The episode of hemolysis observed on POD 8 was considered to be secondary to ECMO circuit obstruction, most likely at the venous drainage side, which resulted in elevated negative pressure within the circuit. Such excessive suction pressure is known to cause mechanical hemolysis and, at the same time, compromise effective ECMO flow. In this setting, inadequate left heart decompression during VA-ECMO can occur, particularly in the presence of residual left atrioventricular valve regurgitation. We postulated that impaired left atrial unloading due to circuit dysfunction led to further elevation of left atrial pressure, thereby aggravating pulmonary congestion and hemorrhage. Therefore, atrial septal defect creation was performed not merely as a response to hemolysis, but as a definitive strategy to restore effective left atrial decompression and stabilize cardiopulmonary dynamics.

However, considering that the primary cause of cardiac arrest was not hemorrhage itself but asphyxia—a potentially reversible condition—the indication for ECMO warrants more proactive consideration. In addition, this patient was in a postoperative open-chest state, which allowed rapid access to the heart and great vessels. This contributed greatly to the prompt initiation of ECMO, although this circumstance also represents a limitation, as such conditions are not common and cannot be generalized to other patients. Although it is plausible that ECMO contributed significantly to survival in this case, a causal relationship cannot be established from a single case report. Regarding neurological outcome, the patient demonstrated no apparent neurological deficits at the time of hospital discharge. Clinical neurological assessment revealed appropriate spontaneous limb movements, preserved feeding ability, and satisfactory weight gain. However, formal neuroimaging studies such as magnetic resonance imaging or computed tomography, as well as standardized neurodevelopmental assessments, were not performed during hospitalization. Therefore, while the short-term neurological outcome was clinically favorable, the absence of formal neurodevelopmental evaluation represents an important limitation of this case, and careful long-term follow-up is warranted.

Furthermore, the balance between anticoagulation and hemostasis during ECMO in the setting of pulmonary hemorrhage must be individualized, and low-intensity or heparin-free approaches have been described in select cases, depending on bleeding severity and underlying pathology (2, 9).

Several similar and contrasting cases have been reported in the literature, including neonatal and pediatric pulmonary hemorrhage managed with ECMO (1, 5–7), as well as cases associated with infection or cardiac disease such as Mycoplasma pneumonia, idiopathic pulmonary hemosiderosis, and vasculitis-related diffuse alveolar hemorrhage 3, 7–9). However, reports of patients who experienced airway obstruction, severe asphyxia, and cardiac arrest before ECMO initiation remain extremely rare (8), underscoring the distinctiveness of the present case.

## Patient perspective

The patient's parents did not directly witness the events. They later recalled experiencing intense anxiety upon learning that ECMO had been urgently initiated. Following the patient's full recovery, they expressed deep gratitude for the medical team's prompt actions.

## Data Availability

The raw data supporting the conclusions of this article will be made available by the authors, without undue reservation.
